# DFU-GCNet: a global context-enhanced inception network for robust and interpretable diabetic foot ulcer classification

**DOI:** 10.3389/fdgth.2026.1811311

**Published:** 2026-05-28

**Authors:** Md. Tofael Ahmed Bhuiyan, Md. Abdur Rahman, Farzan Majeed Noori, Md Zia Uddin, Abdul Kadar Muhammad Masum

**Affiliations:** 1 Computational Intelligence Lab, Southeast University, Dhaka, Bangladesh; 2 Department of Informatics, University of Oslo, Oslo, Norway; 3 Sustainable Communication Technologies Department, SINTEF Digital, Oslo, Norway; 4 Department of Computer Science and Engineering, Southeast University, Dhaka, Bangladesh

**Keywords:** deep learning, DFU-GCNet, diabetic foot ulcer, explainable AI (XAI), global context attention, medical image classification

## Abstract

**Introduction:**

Diabetic foot ulcers (DFUs) are severe complications that cause frequent lower extremity amputations. Timely diagnosis is crucial for effective clinical management. Although deep learning approaches improve detection, the models often struggle to capture different lesion scales. Furthermore, opaque algorithmic decisions often lower medical trust. Therefore, this study introduces DFU-GCNet for robust and interpretable ulcer classification.

**Methods:**

The proposed architecture merges inception modules with global context blocks. This combination extracts multi-scale features from different wound sizes and simultaneously models broad spatial dependencies across tissue regions. Thus, it effectively distinguishes pathology from surrounding healthy skin. We evaluate this framework using the Kaggle DFU dataset. We integrate explainable AI techniques to ensure clinical transparency. GradCAM++, Local Interpretable Model-Agnostic Explanations, and SHapley Additive exPlanations are used to provide high-resolution diagnostic heatmaps and confirm that the network prioritizes clinically relevant wound boundaries.

**Results:**

The model achieved a superior classification accuracy of 97.16%, with an F1-score of 0.9715 and a Matthews correlation coefficient of 0.9437. DFU-GCNet demonstrated decisive superiority compared with standardized modern baselines such as VGG16 and EfficientNet.

**Discussion:**

The findings indicate that DFU-GCNet is a highly reliable automated screening instrument.

## 1 Introduction

Diabetes mellitus is a widespread metabolic disorder that causes chronic hyperglycemia. Untreated hyperglycemia presents severe health risks, including permanent blindness. Cardiovascular diseases, renal failure, and limb loss also manifest (1). Diabetic foot ulcers (DFUs) are a particularly debilitating complication. These chronic, non-healing wounds primarily afflict the lower extremities, lead to severe infections, and drastically compromise patients’ quality of life. In 2014, estimates indicated that almost 422 million individuals were affected globally, with a global adult diabetes prevalence of 8.5% (2). Projections forecast over 600 million global cases by 2035. Developing nations bear nearly 80% of this burden (3). Approximately 15%–25% of patients develop foot ulcers. Without proper care, many require lower extremity amputations (4).

Over 1.5 million diabetic patients undergo amputations (5). These preventable outcomes highlight a critical need for self-care. Essential practices include strict hygiene and medication compliance. Regular clinical checkups are another mandatory healthcare component. Globally, the financial implications of managing diabetes are enormous. In poorer nations, treatment costs frequently exceed low household incomes. Costs can reach 5.7 times the average income (6). This creates an uncontrollable economic burden for affected families. An increase in diabetes prevalence will increase diabetic foot ulcer prevalence (7). This trend will further exacerbate global healthcare challenges. A shortage of skilled medical personnel impedes adequate care. Furthermore, specific preventative infrastructure remains largely non-existent across regions. Consequently, a vicious cycle of inadequate treatment persists. Millions suffer devastating, preventable limb loss from these complications.

Proper clinical assessment requires a meticulous examination of medical records. Manual evaluation processes remain susceptible to human error and inefficiency. Computer-aided diagnostic systems can significantly improve diagnostic accuracy. These systems subsequently reduce process delays and overall healthcare costs. Mobile health technologies are shifting the diabetes care paradigm. They enable the real-time tracking of physiological indicators. These tracked indicators include local foot pressure and inflammation. Early complication detection greatly enhances patient outcomes (8). This detection increases remission longevity and improves life quality. Therefore, an automated diagnostic infrastructure for ulcers is crucial. Such infrastructure must be non-invasive, remotely accessible, and highly reliable. Recent diagnostic developments leverage deep learning and computer vision. Contemporary clinical practice relies on complex diagnostic methodologies. Practitioners thoroughly examine the complete medical history of patients. Advanced medical imaging supplements these specialist physical examinations. This imaging guides interventions by establishing accurate ulcer progression.

Modern biomedical science has progressed at a rapid pace. Researchers have successfully mapped complex protein structures. Enhanced biomedical image classification is another significant achievement. Furthermore, genetic sequence alignment now operates with high precision. Cutting-edge computing technology facilitates these transformational scientific achievements. Efficient computational frameworks are urgently required for continued progress. These systems must process high-dimensional, heterogeneous datasets efficiently. Deep learning acts as a truly disruptive technology here. Neural networks learn complex hierarchical representations from unprocessed data. This seamlessly enables the simulation of complicated biological systems and accurately detects minute patterns crucial for medical diagnosis.

Deep learning methods are therefore vital in modern biology. They process massive datasets to discover clinically significant trends. These advanced approaches greatly assist in accurate disease prognosis. Digital healthcare relies heavily on medical imaging (9). Imaging aids the early diagnosis of pathological disorders (10). Machine learning extracts sensitive visual features from these images. Extracted attributes include specific shape, color, and size features. Previous research confirms model effectiveness in identifying foot ulcers. However, the inherent opacity of deep learning challenges adoption. This black-box property hinders widespread clinical-level implementation. Poor interpretability obscures the identification of underlying computational biases. It also complicates the judgment of potential error sources. Comprehending the reasoning behind model predictions remains quite difficult. These complex algorithms also frequently overfit the training data. Furthermore, they demonstrate extreme hypersensitivity to chosen model hyperparameters. This restricts their generalization to entirely new datasets. Transparent machine learning successfully overcomes these critical implementation issues. It provides direct explanations for previously unexplainable model behavior. This renders diagnostic systems significantly more reliable and responsible. Consequently, operational reliability in sensitive biological setups is enhanced.

Deep learning models have achieved a major breakthrough in the study of ulcers. They are effective in extracting more detailed patterns from medical images. The black box nature of them, however, makes them incompatible with clinical integration. A few major concerns have hindered wide adoption, including potential model biases and insufficient transparency. Other problems include the privacy of data and unresolved liability. Explainable artificial intelligence transforms these unreadable systems down to their foundations. It generates white box models, which are understandable to clinicians. In this way, confidence is increased and productive cooperation is developed. However, the intelligible methods that exist currently tend to be insufficient. They do not provide clinically useful insights on a regular basis. Thus, an ideal solution to complex diagnostic needs is required.

This study presents DFU-GCNet for the accurate diagnosis of diabetic ulcers. The architecture is a combination of inception blocks and improved global context (GC) modules. Parallel processing branches recognize different ulcer patterns at different scales. Global context blocks are good at modeling long-range spatial dependencies properly. Residual connections merge semantic information without losing local texture details. Label smoothing techniques help to prevent overfitting by moderating the target confidence. Data augmentation techniques mimic various clinical lighting and camera angles. This model has a superior accuracy in the Kaggle ulcer dataset. Explainable algorithms are algorithms that can visualize different regions of pixels that affect the final decision. This is a framework that outperforms traditional convolutional networks in terms of robust performance metrics.

To summarize, the main contributions of this study are as follows:

- DFU-GCNet integrates global context blocks for enhanced feature representation.
- Inception modules extract multi-scale features to handle ulcer variations.
- Enhanced residual blocks combine local textures with global semantic information.
- Label smoothing loss functions improve generalization on noisy medical datasets.
- AdamW optimization decouples weight decay to ensure stable model convergence.
- The proposed framework outperforms state-of-the-art architectures in accuracy.
- Interpretability tools such as SHapley Additive exPlanations (SHAP) provide transparent insights for clinical trust.

This article comprises eight sections. The second section is a literature review and an overview of the current deep learning models. The third section describes the suggested network design and required elements. The fourth section will outline training plans and data enhancement streams. The fifth section contains the analysis of comparative performance to benchmarks. The sixth section introduces model interpretability using elaborate visual explanation tools. The seventh section focuses on existing constraints and future research directions. The last eighth section provides the conclusion and highlights findings. This systematic arrangement provides a rational direction for the investigation.

## 2 Related work

Recent studies have proposed multiple categorization schemes for DFU identification. The foundational research underpinning the proposed approach is critically synthesized in this section. Recently, an elaborate analysis was conducted by Kaselimi et al. (11). They reviewed the use of artificial intelligence in ulcer monitoring techniques, elucidating the advantages and inherent implementation challenges. The research dealt with remote healthcare environments in particular. It analyzed particular patient physiological aspects. Communication with sensor characteristics was also determined (12). Imaging and optical sensor technologies were focused on. The study showed that the data source had a critical influence, as the type of data has a direct impact on the choice of the monitoring technique. This then decides the appropriate choice of artificial intelligence algorithm (11).

This is an important area in which Das et al. (13) have contributed significantly. Their new convolutional neural network is called DFU-SPNet. This design compares to the current classification performance standards in the context of high performance (13). The architecture is entirely comprised of a number of sequential convolutional layers. The kernel size in each layer begins with different numbers. Deeper feature extraction is possible due to the transition layers. The activation functions are mainly leakyReLU. Operation package normalization is seamless as well. One disadvantage concerns the filter application. The model employs a set of fixed convolutional filters across all the branches. These filters are arranged in a 32, 64, 128 pattern. A non-homogeneous filter structure may enhance the resilience of the models. Such a method could increase the level of classification significantly.

A hybrid network was proposed by Alzubaidi et al. (14) to classify DFUs. This model is a combination of parallel multi-branched modules with traditional convolution layers. There are four different networks with six parallel convolutional blocks to extract features. The parallel branches can range between two and five through a certain range of convolutional layers. The representational richness of granular feature maps is promoted by a variety of receptive field widths. Parallel modules are complicated and demand a lot of resources and high computational cost. However, despite achieving high accuracy rates, the main limitation of such architectural complexity is its high computational cost. These parallel structures could be refined to enhance the efficiency of the entire process. Further studies have to strike a compromise between performance in classification and the limited hardware performance. Such developments can provide vital information in the creation of diagnostic medical software.

DFU_QUTNet was suggested by Alzubaidi et al. (15) to classify diabetic ulcers. The model maximizes breadth in architecture and maintains depth in networks, which is essential. DFU_QUTNet features are extracted and are trained by traditional machine learning classification algorithms. The F1-score is greatly improved with the support of support vector machine (SVM) and k-nearest neighbors (KNN) classifiers. The proposed framework makes use of 3  ×  3 kernels in all convolutional layers. This uniform kernel set is a limitation to effective multi-scale feature extraction. The difference in the size of the kernel may enhance network generalization and classification accuracy. The next generation needs to have different fields of receptivity to achieve higher performance. The use of multi-scale filters provides a more powerful analysis of the images. These enhancements mitigate current drawbacks in deep learning designs.

Goyal et al. (16) presented DFUNet to identify diabetic foot ulcers. This structure is a combination of the single convolutional stages and parallel convolutional blocks. The Linde–Buzo–Gray (LBG) method has been used as a trusted low-level feature extractor. The LeNet, GoogleNet, and AlexNet models are used to extract high-level features. The features that are extracted are input into special machine learning models. One of the most significant restrictions is the lack of transition layers between parallel units. Multi-layer concatenated feature map extraction could be enhanced by transition layers. Newer designs ought to focus on fine-tuning transitioning structures to achieve better performance. This would probably increase the overall accuracy of classification. These contributions are necessary for diagnostic devices in medical care.

Wang et al. (17) developed a dual-phase classification system for DFUs. This technique is a combination of capture boxes and a support vector machine. The former step determines the localities of interest through superpixel classification. Relevant features are removed in stage 2 of the analysis. This is a two-stage categorization technique that is useful for defining particular zones in diabetic ulcers. Clinical wound monitoring relies on data that are obtained using automated detection systems. The framework proves to be very efficient in detecting the boundaries of a complex lesion. SVM classifiers were used by the researchers to maximize the total prediction accuracy. These innovations enhance the accuracy of clinical instruments. The incorporation of a multi-phase workflow increases the quality of medical imaging.

Goyal et al. (18) proposed a two-scale transfer-learning architecture. Their framework uses fully convolutional networks (FCNs) to examine an entire foot image. The complex method differentiates between diabetic foot ulcers and the adjacent skin areas. An analysis provided a Dice similarity coefficient of 0.851 in the segmentation of DFUs. The mean coefficient score for surrounding skin segmentation was 0.794. The merged stage attained an excellent Dice similarity coefficient of 0.899. The deep learning model is robust because it has high levels of accuracy. Transfer learning is significant in enhancing the quality of medical image segmentation tasks. These FCN model combinations in clinical diagnostic processes can be integrated into future research.

Existing methods possess certain drawbacks despite their impressive accuracy numbers. Managing large datasets remains problematic and impedes practical scaling efforts. Physical contact during data collection raises serious clinical infection concerns. Earlier research often lacks crucial explanation and transparent interpretation qualities. Opaque deep learning architectures prevent necessary trust among medical practitioners. The proposed study aims to resolve these intrinsic architectural shortcomings. To further contextualize our proposed DFU-GCNet within the rapidly evolving landscape of medical image analysis, it is imperative to acknowledge recent advancements employing vision transformers (ViTs) and ensemble architectures. For instance, recent studies have explored attention-driven ViT models to capture global dependencies in wound images, albeit often requiring significantly larger computational overhead compared to convolutional neural networks (CNNs) (19). Similarly, ensemble methods and multimodal sensing systems have demonstrated improved generalization and real-time applicability in resource-constrained clinical settings (20). Furthermore, the integration of multimodal data, such as combining thermal imaging with standard RGB clinical photographs, has emerged as a promising avenue to enhance early-stage ulcer detection accuracy, especially with recent advances in thermal image correction techniques (21). While these approaches offer distinct advantages, our DFU-GCNet framework maintains a highly efficient parameter footprint by synergizing inception modules with global context blocks, providing an optimal balance between computational efficiency, high predictive performance, and robust interpretability. Table 1 provides a comprehensive overview of pertinent published research.

**Table 1:** Existing approaches for DFU detection.

Article	Dataset	Outcomes/strengths	Weaknesses
Das et al. (13)	3,827	Utilized stacked parallel convolution layers with suitable transition layers (TLs)	Repeated use of the same filter pattern (32, 64, and 128) for each PCB
Alzubaidi et al. (14)	17,053	Combined traditional convolution layers with multi-branch parallel convolution layers	High computation time
Alzubaidi et al. (15)	17,053	Enhanced model width while reducing computing cost	Uniform filter sizes (3 × 3) for PCB
Goyal et al. (16)	22,777	Reduced model depth but increased filter sizes in PCB	Insufficient transition layers between parallel convolution blocks
Wang et al. (17)	100	Identified wound areas using a capture box and an SVM classifier	Slightly slow running speed
Goyal et al. (18)	705	Predicted pixel-level segmentation for DFU samples	Limited dataset size

While the aforementioned state-of-the-art methods demonstrate significant progress in automated DFU detection, a critical analysis of these architectures reveals three primary research gaps. First, models utilizing uniform or rigid filter patterns (13, 15) inherently struggle to capture the extreme multi-scale morphological variations typical of diabetic ulcers. Second, architectures that attempt to solve this via highly complex parallel branching (14) incur steep computational costs, limiting their practical deployment in resource-constrained or mobile clinical environments. Finally, and most critically, nearly all existing SOTA approaches lack integrated interpretation mechanisms. This “black-box” opacity prevents necessary trust among medical practitioners and leaves it unclear whether models are learning genuine pathological features or simply memorizing confounding background artifacts. The proposed DFU-GCNet explicitly addresses these gaps by synergistically combining multi-scale inception modules to handle ulcer size variations, lightweight global context blocks to efficiently model long-range spatial dependencies, and a comprehensive explainable AI (XAI) suite to ensure total diagnostic transparency.

## 3 Methodology

**Figure 1:**
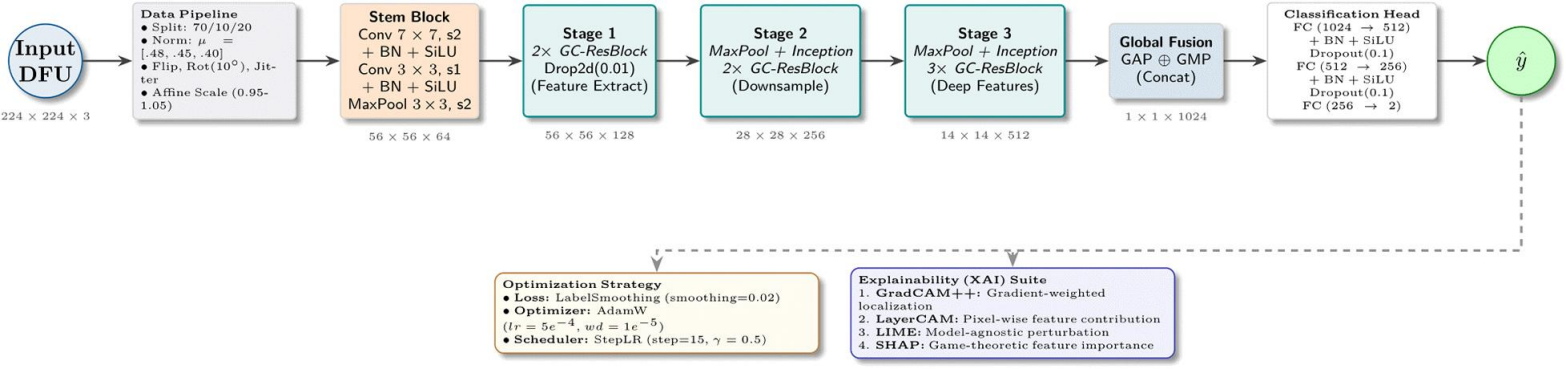
Schematic diagram of the proposed DFU-GCNet architecture pipeline.

The proposed DFU-GCNet processes 224 × 224 pixel inputs using enhanced stem structures. Initial 7 × 7 and 3 × 3 convolutions extract robust features. Global context blocks model long-range spatial dependencies via attention mechanisms. Four parallel inception branches capture multi-scale ulcer features during processing. Residual blocks with 0.01 dropout integrate local and global information properly. Three distinct stages utilize 128, 256, and 512 feature channels, respectively. Dual pooling strategies generate 1,024 dimensional vectors for final classification tasks. Three fully connected layers produce predictions for two distinct target classes. The AdamW optimizer employs 1×10^−5^ weight decay for model training stability. Data augmentation includes 10° rotations and 0.02 label smoothing techniques. Gradient-weighted class activation mapping (Grad-CAM++), local interpretable model-agnostic explanations (LIME), and SHAP provide pixel-level importance for clinical model validation. This architecture ensures transparency and high performance for medical diagnostic tools. A step scheduler reduces the learning rate every 15 training epochs. Figure 1 shows the proposed DFU-GCNet architecture pipeline.

### 3.1 DFU dataset

The dataset used in this work comes from four different folders on the Kaggle online data repository (15), and its origins are explained in previous research (22). In particular, information was gathered from the Diabetic Department at Nasiriyah Hospital, which is situated in southern Iraq (15). All participants' informed permission was properly acquired, and ethical approval was sought due to the ethical concerns surrounding medical imaging data, especially those that include sensitive patient information. To represent variation in the real world, the dataset includes clinical foot images taken with consumer-grade photographic equipment, including iPads and Samsung Galaxy smartphones, in a range of lighting and configurations. In order to create and assess the model, this research concentrated on the 1,055 segmented skin image patches that make up the “patch” subset of the dataset. In total, 543 cases were classified as non-ulcerated (healthy skin), while 512 cases were clinically verified as ulcerated (abnormal). While the dataset contains a minor class imbalance (543 normal vs. 512 ulcerated cases), this was strictly managed during the experimental design through stratified data splitting, ensuring proportional representation across all training, validation, and testing partitions. To prevent this slight imbalance from skewing performance interpretation, our primary evaluation heavily weights imbalance-aware metrics such as the Matthews correlation coefficient (MCC) and Cohen's Kappa.

### 3.2 Stem and initial feature extraction

The stem module processes 224 × 224 input images efficiently. Initially, a 7 × 7 convolutional layer with a stride of 2 is applied to the input. This initial operation captures low-level edges and spatial features. A 3 × 3 convolution then increases early network capacity. This layer derives non-linear mappings in a very complex manner from the raw pixels. After every convolution, batch normalization is used to stabilize every internal activation. The SiLU activation function provides a beneficial gradient flow during the training process. The max pooling layer is a three-facet downsizing layer that decreases the spatial dimensions. This pooling step is strictly a stride of 2. The module holds significant textural information for the advanced phases. High-quality inputs in stage 1 are refined feature representations. The first part of feature extraction is preserved in terms of computational efficiency. This sound stem framework makes data available for modeling on a global context. Each architectural decision maximizes the initial processing of ulcer images. The generated feature maps include vital classification data.

**Figure 2:**
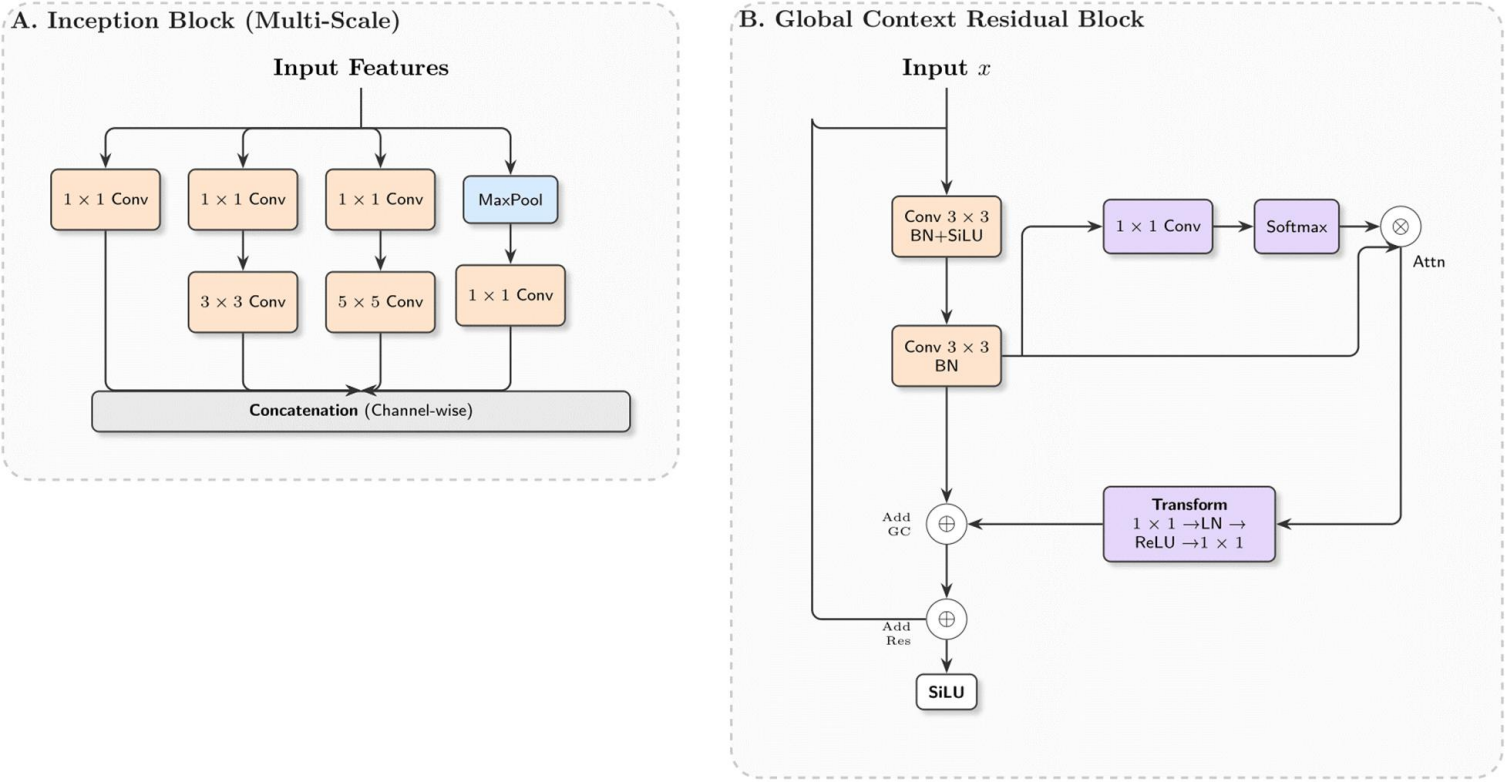
Structure of the **(A)** inception and **(B)** global context-enhanced residual blocks.

Internal engineering of the two building blocks of the network is described in Figure 2. In section A, there is the inception block, which employs four parallel branches with different kernel sizes (1 × 1, 3 × 3, and 5 × 5) to capture multiple-scale ulcer features at once. Section B gives an explanation of the global context residual block, which separates the context modeling, feature transformation, and fusion stages. This scheme represents how the mechanism combines global spatial dependencies through attention masks and integrates them with local features. The figure illustrates how the particular components allow the model to execute complicated biological textures and ensure computational efficiency through residual learning.

### 3.3 The global context block

One of the fundamental architectural innovations is the global context block. This module facilitates overall knowledge of diabetic foot ulcer images. The convolutional standard operations have local receptive fields. The GC block is used to summarize the complete spatial feature map. It is a combination of non-local networks and squeeze-and-excitation modules. The block uses an input feature map, which is denoted by X. The dimensions of this input are batch by channel by height by width. The block has three consecutive steps, namely, context modeling, feature transform, and fusion. The context modeling is first done by computing a global spatial attention mask. A channel convolution with one dimension is used to reduce the channel dimension. A softmax is subsequently performed in all spatial locations. This produces a normalization of attention per location.

The attention weight alpha at position *j* is calculated using Equation (1):

$$\alpha_{j}=\frac{e^{W_{k}x_{j}}}{\sum_{m}e^{W_{k}x_{m}}}$$ (1)

Here, *x_j_* represents the feature vector at spatial index *j*. The matrix *W_k_* denotes the one-by-one convolutional weight. The computed attention map captures the relative spatial importance globally. A compact global context vector Z is subsequently formed. It is a weighted sum of all input features using these attention weights, as formulated in Equation (2) as follows:

$$Z\;=\;\overset{N}{\underset{\;j=1}{\sum }}\alpha_{j}x_{j}$$ (2)

The second step is a channel-wise feature transformation. This is a model that interdepends on feature channels. *Z* is a context vector that is sent through a bottleneck structure. The channel dimension is then reduced by a ratio, r, first through a one-by-one convolution. The distribution of the reduced features is stabilized by layer normalization. Non-linearity is necessary and is added through a ReLU activation function. The original number of channels is restored using a final one-by-one convolution.

This transformation is mathematically expressed in Equation (3) as follows:

$$\delta (Z)=W_{v2}\;\mathrm{ReLU}!(\mathrm{LN}!(W_{v1}Z))$$ (3)

The final step is the fusion of the global context with local features. The transformed context delta of *Z* is added to the original input *X*. This fusion uses a residual-style element-wise addition operation, defined by Equation (4), as follows:

$$Y_{i}=X_{i}+\delta (Z)$$ (4)

This addition injects global semantic information into local representations. The original local texture details are simultaneously preserved. The entire block is computationally lightweight yet highly effective. It provides the model with global vision at critical processing stages.

#### 3.3.1 Context modeling

The context modeling step is used to build a global spatial attention map. This map marks out areas of relative significance in feature volumes and 1:1 convolution downsizes the number of input channels. The linear projection produces a one-channel spatial significance map. Each of the points in this map is characterized by a scalar value. All the values at these spatial positions are normalized using softmax. This normalization will make the attention weights add to unity. The weight distribution highlights areas of features with global significance in an extremely powerful manner. This model expresses long-range dependencies in the entire image. The softmax operation focuses on the features for which the activation is much higher. It gives a calculation on a probability distribution of all available spatial locations. The network is able to learn which pixels contribute the most to the global context. This process connects the ulcer area to the adjacent healthy area. The context feature *Z* is both a localized and a global feature. This is the end of the context modeling of this particular block.

#### 3.3.2 Feature transform

The transform step is a critical operation designed to capture complex inter-channel dependencies within the aggregated global context vector, *Z*. To strictly govern model complexity and mitigate the inherent risk of overfitting associated with relatively constrained medical datasets, this operation is executed through a highly constrained bottleneck architecture. Initially, the channel dimension of the context vector undergoes a massive dimensionality reduction via a 1 × 1 convolutional layer, utilizing a reduction ratio of *r* = 16. This structural bottleneck restricts the proliferation of learnable parameters, forcing the network to learn a compact, distilled representation of informative features rather than memorizing noise.

The complete transformation is mathematically defined in Equation (5) as follows:

$$\delta (Z)=W_{v2}\;\mathrm{ReLU}!(\mathrm{LN}!(W_{v1}Z))$$ (5)

Following this compression, layer normalization (LN) is applied to the reduced feature vector. Beyond counteracting internal covariate shift and balancing activation distributions, LN acts as an implicit regularizer, promoting stable convergence independently of batch size constraints. Essential non-linearity is subsequently introduced via a ReLU activation function, enabling the assimilation of complex, hierarchical representations without expanding the parameter space. Finally, a subsequent 1 × 1 convolutional layer reconstructs the tensor back to the original input channel dimension, *C*. When coupled with the network's global regularization strategies, specifically AdamW decoupled weight decay, label smoothing, and aggressive stochastic data augmentation, this bottleneck mechanism ensures that the enhanced representational capacity of the transform step does not compromise the model's generalization integrity.

#### 3.3.3 Fusion

The fusion stage is the integration of the transformed global environment into the localities. It is an operation involving a simple but powerful element-wise addition. The former is the refined global context signal delta, *Z*. The second input is the initial input feature map, *X*. The channel and spatial dimensions of each of the two tensors are the same. The last output feature map, *Y,* is their direct addition.

The fusion stage is mathematically expressed by the following equation:

$$Y_{i}=X_{i}+\delta (Z)$$ (4)

*i* in such a formulation refers to an appointed location in space, i.e., a channel. This is added to each of the elements in the volume of the feature in the same manner. A definite gradient pathway is provided by such a residual-type fusion. It facilitates the network's integration of local and global information. Local texture information is an original convolution and this is fully preserved. Meanwhile, semantic relationships across the world are incorporated in the representation. The model also learns to match the character attributes of the ulcers with their context. This is vital in the distinction of mild pathological differences. There are no additional parameters learnt in the operation, which maintains the computational efficiency of the overall block. The product, *Y*, obtained retains the rich color of the original feature map, *X,* and has also been filled with a detailed context. This kind of combined representation is applied to subsequent layers in the network. The global context block, thus, offers greater discrimination power. The whole information integration process is brought out by the combination.

### 3.4 The inception block

The inception block considers extreme variation in the appearance of ulcers. It possesses four pathways that receive input features parallel to each other. These parallel ways use different convolutional kernels in feature extraction. The multi-scale outputs are added up along the specific output channel dimension. The former is based on a convolution that is one-by-one. Branch number 2 has a 3 × 3 successive convolution. The third line utilizes 5 × 5 kernels in order to utilize the space. This specific pathway encompasses gigantic ulcer structures and regional features. The final branch has three pooling operations. This model enables the identification of the various pathological structures with the help of this structure.

The SiLU activation is on the convolutional layers of each block. This ensures gradient flow and homogeneity of optimization. The final product is a sum of all four branches, channel by channel. Such concatenation can be explicitly described by Equation (6) as follows:

$$\mathrm{Output}\;=\;\mathrm{Concat}(\mathrm{Branc}h_{1},\;\mathrm{Branc}h_{2},\;\mathrm{Branc}h_{3},\;\mathrm{Branc}h_{4})$$ (6)

The feature map that is obtained is multi-scale in nature. It is an excellent mixture of minor details and the broader tendencies of the context. This design provides the ulcer size and shape change with intuitive strength. The position of the block is good in the second and third stages of the network. It assists the model in arriving at a comprehensive hierarchical interpretation.

### 3.5 Global context-enhanced residual blocks

The core feature extraction mechanism of our network relies on enhanced residual blocks. We have modified the standard residual function, *F*(*X*), to explicitly incorporate the GC mechanism, thereby improving the model's ability to fuse local textures with broader semantic information. To clarify this architecture, the block's operation is divided into two sequential stages.

#### 3.5.1 Residual pathway redesign

The transformation begins with a 3 × 3 convolutional layer, which is immediately followed by batch normalization and a SiLU activation function. To prevent overfitting without heavily disrupting the information flow, a lightweight dropout layer with a marginal probability of 0.01 is applied. Subsequently, a second 3 × 3 convolution and batch normalization step refine the features. This sequence is specifically designed to capture the fine-grained, local textural details of the ulcer.

#### 3.5.2 Semantic fusion and identity mapping

Following local feature extraction, the intermediate feature maps are fed into the global context block. This module assimilates global semantic dependencies and integrates them into the local pathway. The final transformed signal is then added to the original input block, *X*, via an identity shortcut connection. The complete enhanced residual transformation is mathematically modeled in Equation (7) as follows:

$$Y=X+\;GCBlock(BN(\mathrm{Con}v_{3\times 3}(\mathrm{Dropout}(SiLU(BN(\mathrm{Con}v_{3\times 3}(X)))))))$$ (7)

This synergistic design ensures the simultaneous preservation of local texture and global context. Furthermore, the identity shortcut connection guarantees stable gradient flow during deep network training, yielding a highly expressive and robust computational structure.

### 3.6 Macro-architecture and staging

The DFU-GCNet macro-architecture is designed in three processing stages. These processes gradually change the spatial and semantic feature representations. The successive stages deepen the channel and decrease the spatial resolution. Such a hierarchical structure constructs multi-scale meaning. The network uses input images that have a resolution of 224 × 224.

The high-resolution features of the stem are directly refined in stage one, with a rather shallow channel depth of 128. The step involves the unification of low-level edges and textures. Stage two starts with a down-sampling operation, namely, max-pooling. This enriches the channel depth up to 256 representations. The stage incorporates a single inception block for multi-scale capture. It then uses two global context-enhanced residual blocks. This combination switches between low-level and mid-level abstractions.

Stage three starts with a final spatial down-sampling operation. It also adds a depth capacity of 512 to the channel capacity. This step is a repetition of a particular pattern in the extraction of deep features. This scheme consists of an inception block and three enhanced residual blocks. This bottom-up level is a manifestation of important upper-level semantic concepts in pathology. The entire staging architecture can be characterized by Equation (8) as follows:

$$\mathrm{Input}\;\to \;\mathrm{Stem}\;\to \;{\mathrm{Stage}}_{1}^{C=128}\;\to {\;\mathrm{Stage}}_{2}^{C=256}\;\to \;{\mathrm{Stage}}_{3}^{C=512}\;\to \;\mathrm{Head}$$ (8)

The channel depth of a given stage, in this case, is denoted by the superscript *C*. The staged design will render the distribution of computational resources productive. The early stages preserve the spatial data to identify the location of particular areas. The subsequent stages develop well-developed semantic features to categorize images effectively. The modeling capacity and the representation power are provided by such a structured approach.

### 3.7 Feature aggregation and classification head

The final convolutional layer yields rich and high-dimensional feature maps. This spatial information is summarized into a hybrid dual-pooling strategy. It has global average pooling, which is used in the entire space. This process calculates the average activation of all the feature channels and encapsulates the overallness and strength of the acquired models. The same feature maps are max-pooled globally. This filters the highest activation in each channel and recognizes the most distinguishing and discriminating local evidence.

The outputs from both pooling operations are then concatenated. This forms a comprehensive one-dimensional feature vector, *V*_final_. The concatenation process is formally defined in Equation (9) as follows:

$$V_{\mathrm{final}}\;=\;\mathrm{Concat}(\mathrm{GlobalAvgPool}(X),\;\mathrm{GlobalMaxPool}(X))$$ (9)

The architecture is 1,024 in size. A three-layer fully connected classifier is used to pass this vector. The initial linear layer decreases the size (1,024) by half (512). This linear transformation is followed by batch normalization and SiLU activation. To regularize it, a dropout layer with a rate of 0.1 is used. The second linear layer further downsizes the dimension from 512 to 256. It also has batch normalization, SiLU activation, and dropout. The last linear layer projects the feature to the number of classes. This layer directly gives the raw classification logits. Internal covariate shift is alleviated using batch normalization in fully connected layers. The minimal dropout rate encourages an ensemble behavioral pattern during training. This head design guarantees strong and understandable final predictions.

## 4 Training strategy and optimization

The proposed model encompassed intensive medical imaging methodology. There was an extensive data augmentation pipeline based on geometric and photometric transformations. Randomly flipping and rotating were used to simulate different angles and camera orientations. Scale and distance variance, perspective variance with affine translations, brightness, contrast, and color jitter gave the effect of varying light conditions. Intensity of augmentation was strictly controlled such that the pathological aspects of the underlying condition were not lost. The loss used labeled smoothing rather than cross-entropy. In order to avoid overconfidence, label smoothing was used to substitute hard one-hot target vectors. AdamW was chosen to decouple the weight decay. This offers better regularization than the regular Adam optimizer. The initial learning rate was set to 0.0005. A step learning rate scheduler decreased the rate every 15 iterations. This was a strategy that enabled quick exploration and accurate convergence. The integrated methodology provided precision and good model performance. The approach guarantees that the generalizability is high in different clinical diagnostic settings.

**Figure 3:**
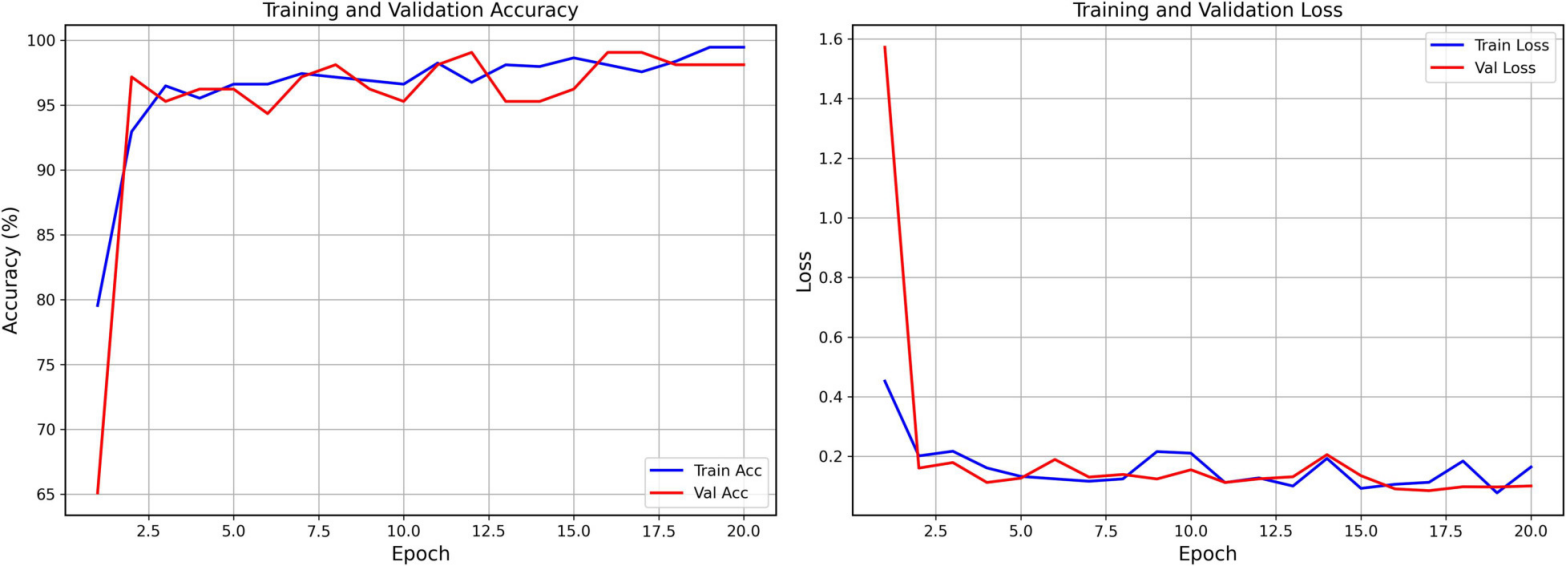
Training and validation accuracy and loss curves during optimization.

Figure 3 shows the quantitative training of the DFU-GCNet with 20 epochs. The left figure indicates the accuracy of the classification, which has a steep increase and then levels off in both the training and validation sets, which means that it learned successfully. The graph on the right shows the loss curves with a consistent downward curve, which proves that the AdamW optimizer and the label smoothing loss function were effective in reducing error. The training and validation lines were very close, indicating that the model generalized well and did not overfit. This visual evidence proves the consistency of the optimization method and that the learning rate scheduler worked.

## 5 Data augmentation

Healthcare data need to be enhanced with enough vigor to guarantee the model generalization. This is a complex pipeline using both geometric and photometric transformations. Random horizontal and vertical flips are used to represent a clinical camera’s different perspectives. To preserve the anatomical position, only 10° of rotation were allowed. To adapt to distance, affine transformations such as scaling and translation were used. Color jitter varied brightness and contrast in reasonable biological ranges. Saturation and hue changes were used to recreate different clinical lighting conditions. The intensity was adjusted to retain all the relevant pathological ulcer characteristics. Changes were implemented in a stochastic manner within every single training epoch cycle. The method increases the diversity of the datasets and enhances invariant visual patterns. The 0.02 epsilon label smoothing eliminated overconfidence errors in model prediction. The augmentation pipeline was invariant-guaranteed, and diagnostic content was not transformed. The entire training setting and parameters are given in Table 2. Optimized pipelines eliminated overfitting of deep networks. Stochastic variations produced different images each time the training was run.

**Table 2:** Parameter values used in the DFU-GCNet framework during training.

Parameter	Value
Input image size	224 × 224 pixels
Batch size	32
Epochs	20
Images per batch	32
Learning rate	0.0005
Weight decay (global L2)	1 × 10^−5^
Optimizer	AdamW
Loss function	Cross-entropy with label smoothing
Label smoothing factor	0.02
Dropout rate (residual blocks)	0.01
Dropout rate (classifier head)	0.1
Early stopping patience	Eight epochs
LR scheduler type	StepLR
LR decay factor (Gamma)	0.5
LR decay step size	Every five epochs
Training/validation/test split	70%/10%/20%

### 5.1 Loss function: label smoothing

The common cross-entropy loss is applied using hard one-hot target vectors. Excessive confidence in models can be stimulated by this formulation. The effect of overconfidence is usually overfitting to noisy medical data. Label smoothing overcomes this important weakness. It substitutes the hard target distribution with the soft one. The new target of a particular true class *k* is computed. A little smoothing parameter epsilon is included in this calculation. All the experiments were to be conducted with a value of epsilon of 0.02. *K* represents the total number of classes.

The smoothed target for class k is computed using Equation (10) as follows:

$$y_{k}^{LS}\;=\;y_{k}(1\;-\;\varepsilon )\;+\;\frac{\varepsilon }{K}$$ (10)

In this equation, *y_k_* represents the original one-hot encoded label. The former is a scaling of the actual class likelihood by (1−ε). In the second term, the probability mass of all classes equals epsilon. This forms a probabilistic aim that deters absolute certainty. The model is thus unable to draw infinite logits to the accurate class. It rather learns less biased and stronger feature representations. This method is an effective way to regularize. It punishes the model for growing too complacent in training. The loss landscape is more navigable and smoother. The clusters of features are further condensed and differentiated. There is better generalization performance, particularly on borderline or ambiguous cases. Label smoothing is a simple yet highly effective modification. It significantly enhances model reliability for clinical diagnostic tasks.

### 5.2 Optimizer and scheduling

The AdamW optimizer was employed for all model training procedures. AdamW modifies the standard Adam optimization algorithm significantly. It decouples the weight decay term from the gradient update step. This correction provides more effective regularization during training. The update rule for a parameter *θ* at time *t* is shown in Equation (11) as follows:

$$\theta_{t}\;=\;\theta_{t-1}\;-\;\eta \left(\frac{\hat{m_{t}}}{\sqrt{\hat{v_{t}}}\;+\;\varepsilon }\;+\;\lambda \theta_{t-1}\right)$$ (11)

Here, *η* represents the learning rate, set to 0.0005 initially. The terms $\hat{m}$ and $\hat{v}$ are bias-corrected momentum estimates. The constant epsilon ensures numerical stability in the denominator. The hyperparameter lambda controls the weight decay magnitude. Weight decay was consistently set to a value of 1 × 10^−5^.

A step learning rate scheduler managed the training progression. This scheduler reduced the learning rate by a fixed factor gamma. The gamma value was configured at 0.5 for the experiments. The reduction occurred periodically every step size number of epochs. The step size was set to five epochs throughout the training phase. The scheduled learning rate at epoch e is formally defined by Equation (12) as follows:

$$\eta_{e}=\eta_{0}\cdot \gamma^{e/s}$$ (12)

In this equation, $\eta_{0}\;$ denotes the initial learning rate. The variable *s* represents the step size of five epochs. The discrete reduction schedule is controlled by the floor function. This plan enables investigations of the loss landscape fast and early. It then allows fine-tuning of convergence to be accurate. The combination guarantees the stable and efficient optimization of models.

### 5.3 Experimental evaluation metrics

The model's performance was rigorously assessed using a comprehensive suite. Evaluation occurred on a stratified 20% hold-out test set. This ensured a fair and unbiased estimation of generalization capability. The assessment prioritized metrics robust to class imbalance. Standard classification accuracy was reported as the primary baseline. Precision and recall were computed independently for each class. The harmonic mean of precision and recall yields the *F*1-score, as calculated in Equation (13) as follows:

$$F1\;=\;2\;\times \;\frac{\mathrm{Precision}\;\times \;\mathrm{Recall}}{\mathrm{Precision}\;+\;\mathrm{Recall}}$$ (13)

The Matthews correlation coefficient provided a more informative statistic. MCC calculates a correlation between predictions and true labels. It is defined using true and false positive and negative counts, as shown in Equation (14):

$$MCC\;=\;\frac{TP\;\times \;TN\;-\;FP\;\times \;FN}{\sqrt{\left(TP+FP)(TP+FN)(TN+FP)(TN+FN\right)}}$$ (14)

This coefficient returns a value between negative one and positive one. It is considered highly reliable for imbalanced medical datasets. Cohen's Kappa statistic measured inter-rater agreement. It accounts for the likelihood of agreement occurring by chance alone and was measured by Equation (15) as follows:

$$\kappa \;=\;\frac{\;p_{o}\;-\;p_{e}}{1\;-\;p_{e}}$$ (15)

Here, *p_o_* represents the observed agreement probability between raters. The term *p_e_* denotes the expected agreement probability under randomness. The area under the receiver operating characteristic curve was also computed. This metric summarizes the model's discriminative power across thresholds. Macro and weighted averages aggregated per-class metrics holistically. This multi-faceted evaluation ensured a complete performance profile.

## 6 Comparative performance analysis

This section provides a detailed comparative analysis with benchmark models. All the architectures were evaluated in standardized test conditions. The performance measures were diagnostic reliability and clinical applicability. The results indicate the superiority of the proposed DFU-GCNet framework.

### 6.1 Class-specific performance evaluation of DFU-GCNet

The DFU-GCNet model had very high predictive performance. The identification of ulcer regions was accurate, as confirmed by the high precision values. High scores in recall skills denoted strong sensitivity to pathological characteristics. A balanced *F*1-score represented harmonious precision–recall trade-offs. The classification accuracy was 0.9716 on the independent test set. Macro-averaged measures also indicated balanced performance within classes. The entire per-class performance profile is outlined in Table 3.

**Table 3:** Detailed performance results of the DFU-GCNet model.

Class or metric	Precision	Recall	F1-score
Normal	0.9558	0.9908	0.9730
Ulcer	0.9898	0.9510	0.9700
Accuracy	0.9716		
Macro average	0.9728	0.9709	0.9715
Weighted average	0.9722	0.9716	0.9715
MCC	0.9437		
Cohen’s Kappa	0.9430		

The Matthews correlation coefficient was 0.9437. This implies that the relationship between predictions and true labels was very strong. Cohen’s Kappa coefficient was 0.9430. This is a confirmation of considerable consensus, exceeding the chance anticipation. These two measures confirmed the resistance of the model to class imbalance. Clinical reliability and diagnostic consistency were high in the performance profile.

**Figure 4:**
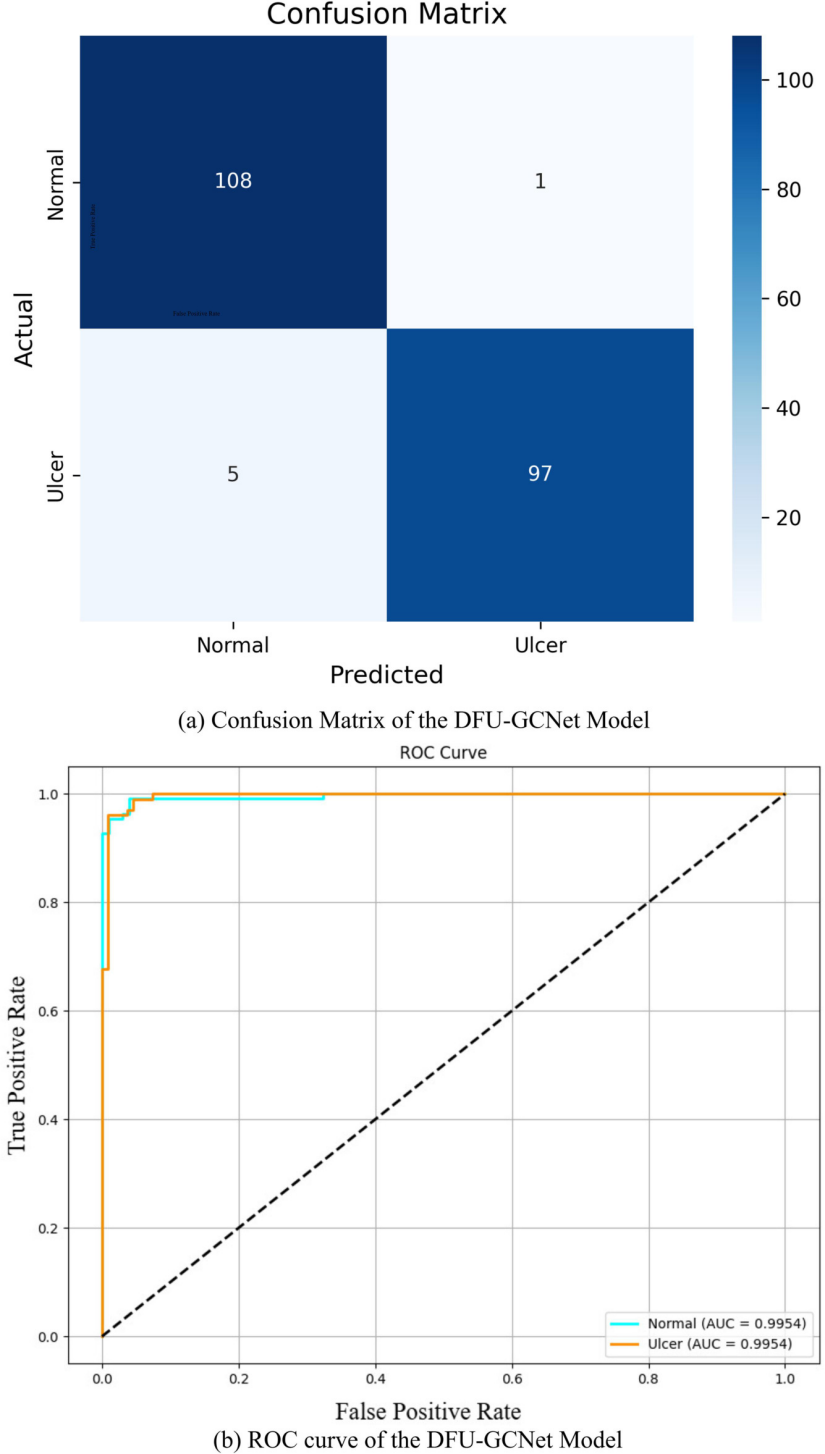
Confusion matrix and receiver operating characteristic curve performance analysis. **(a)** Confusion matrix of the DFU-GCNet model. **(b)** ROC curve of the DFU-GCNet model.

Figure 4 presents a statistical evaluation of the model’s diagnostic reliability using the test data. The confusion matrix is presented in panel (a), indicating the number of true positives and the number of true negatives, with a low misclassification rate between normal skin and ulcers. The ROC curve is shown in panel (b), which is a plot of sensitivity vs. specificity. The curve follows the top-left corner and the AUC is approximately 0.99, indicating near-perfect discriminative ability. All these metrics confirm the validity of the model and its applicability to clinical screening scenarios where it is crucial to be as precise as possible.

### 6.2 Comparative analysis with state-of-the-art architectures

The proposed model was compared against eight well-established deep learning architectures. To ensure a fair and rigorous comparison, all the baseline models were retrained from scratch using the exact same experimental configuration applied to the proposed DFU-GCNet. This standardized setup includes identical data augmentation pipelines, the AdamW optimizer with a 1 × 10^−5^ weight decay, a scheduled learning rate starting at 0.0005, and a cross-entropy loss function with a 0.02 label smoothing factor. A comprehensive summary of this standardized comparative analysis is provided in Table 4.

**Table 4:** Comparative analysis of all the models.

Model	Accuracy	Macro recall	Macro precision	Macro F1	MCC	Cohen’s Kappa
ResNet18	0.8436	0.8467	0.8562	0.8429	0.7028	0.6889
DenseNet121	0.8673	0.8684	0.8687	0.8673	0.7371	0.7350
Xception	0.8910	0.8939	0.9021	0.8906	0.7959	0.7830
EfficientNet	0.9194	0.9208	0.9214	0.9194	0.8422	0.8391
ShuffleNet	0.9336	0.9352	0.9363	0.9336	0.8715	0.8676
MobileNet	0.9479	0.9477	0.9480	0.9478	0.8956	0.8956
VGG19	0.9573	0.9584	0.9584	0.9573	0.9168	0.9148
VGG16	0.9621	0.9627	0.9622	0.9621	0.9248	0.9242
DFU-GCNet	**0**.**9716**	**0**.**9709**	**0**.**9728**	**0**.**9715**	**0**.**9437**	**0**.**9430**

Bold values indicate the best performing metrics across the evaluated models.

Under the standardized training setup, previous discrepancies in model performance were resolved. Foundational networks, such as ResNet18 and DenseNet121, demonstrated baseline capabilities, achieving accuracies of 84.36% and 86.73%, respectively. Modern convolutional architectures, including Xception and EfficientNet, yielded highly competitive results under optimized hyperparameters, reaching accuracies of 89.10% and 91.94%. Lightweight networks, such as ShuffleNet and MobileNet, also adapted well to the task, achieving robust accuracies exceeding 93%. The VGG family was a remarkably powerful traditional baseline for this dataset, with VGG16 attaining an impressive accuracy of 96.21% and an *F*1-score of 0.9621.

Despite the improved performance of the baselines under equitable training conditions, the proposed DFU-GCNet consistently achieved the highest performance across all evaluation metrics. It decisively outperformed the strongest baseline (VGG16), reaching an accuracy of 97.16% and a macro *F*1-score of 0.9715. Furthermore, the substantial improvements in the Matthews correlation coefficient (0.9437) and Cohen's Kappa (0.9430) highlight the model's superior resistance to dataset imbalance and exceptional consistency in inter-class agreement. These findings firmly validate that the architectural developments incorporated into DFU-GCNet, namely the synergy of multi-scale inception features and global context attention, provide a distinct and measurable advantage for diabetic foot ulcer classification.

### 6.3 Discussion on performance superiority and architectural impact

The architectural innovations are important and the performance results are positive. Global context modeling comprises crucial long-range spatial dependencies. This mechanism correlates the features of ulcers with the context of the surrounding healthy tissue. Variable ulcer sizes are dealt with in inception-based multiscale extraction. Parallel convolutional lines extract fine and macro-level details. Stable gradient flow is provided by residual learning between deep network layers. The combination of all these elements in a synergistic manner leads to high performance. The model has the effect of adequately balancing representational power and generalization ability. The results make DFU-GCNet an innovative standard in automated ulcer classification. The framework has great potential to provide reliable clinical decision support.

### 6.4 Cross-validation and statistical significance analysis

To ensure the model's performance was invariant to the specific data split and to definitively rule out overfitting on the limited dataset, a rigorous five-fold cross-validation was executed. As detailed in Table 5, the model exhibited exceptionally stable performance across all folds, yielding a cross-validation mean accuracy of 0.9870 with an extraordinarily low standard deviation of ±0.0049.

**Table 5:** Five-fold cross-validation performance metrics.

Evaluation phase	Accuracy	Precision	Recall	F1-score	MCC	Kappa
Fold 1	0.9822	0.9759	0.9878	0.9818	0.9645	0.9645
Fold 2	0.9941	1.0000	0.9878	0.9939	0.9882	0.9882
Fold 3	0.9822	0.9647	1.0000	0.9820	0.9651	0.9645
Fold 4	0.9882	1.0000	0.9756	0.9877	0.9766	0.9763
Fold 5	0.9881	0.9878	0.9878	0.9878	0.9762	0.9762
CV mean	0.9870 ± 0.0049	0.9857 ± 0.0154	0.9878 ± 0.0086	0.9866 ± 0.0050	0.9741 ± 0.0098	0.9739 ± 0.0099
Held-out Test	0.9812 ± 0.0089	1.0000 ± 0.0000	0.9612 ± 0.0183	0.9801 ± 0.0095	0.9631 ± 0.0172	0.9622 ± 0.0179

To rigorously validate the superiority of the proposed model over the strongest baseline (VGG16), statistical tests were conducted. For the five-fold cross-validation, we employed both the Wilcoxon signed-rank test and a paired *t*-test (*α* = 0.05). Despite the low statistical power inherent in small sample sizes (*N* = 5), the Wilcoxon test achieved its minimum possible *p*-value (*p* = 0.0312) for accuracy, precision, MCC, and Kappa, indicating strict outperformance. The paired *t*-test confirmed statistically significant improvements for recall (*p* = 0.0446) and *F*1-score (*p* = 0.0208). Furthermore, an independent Welch's *t*-test was conducted on the bootstrapped held-out test set (*N* = 1,000 iterations) to evaluate generalization. The proposed model demonstrated highly significant improvements across all metrics (*p* < 0.001), firmly establishing its robust predictive supremacy.

### 6.5 Robustness under noise and adversarial conditions

Clinical environments frequently introduce image degradation due to poor sensor quality or inconsistent lighting. To evaluate the framework's reliability, the trained model was subjected to severe data perturbations, specifically Gaussian noise and fast gradient sign method (FGSM) adversarial attacks.

As outlined in Table 6, injecting high-intensity Gaussian noise (standard deviation = 0.60) only marginally degraded accuracy to 0.9668, proving the model does not over-rely on fragile, high-frequency textural artifacts. Furthermore, under targeted gradient-based adversarial attacks (FGSM at epsilon = 0.02), the model sustained an impressive accuracy of 0.9621. This substantial resilience to adversarial perturbations confirms that the integrated global context attention mechanisms successfully force the network to learn robust, medically relevant morphological structures rather than easily corrupted pixel-level patterns.

**Table 6:** Robustness evaluation under noise and adversarial conditions.

Experiment	Setting	Accuracy	Precision	Recall	F1-score	MCC	Kappa
Gaussian noise perturbation	Std Dev = 0.60	0.9668	0.9672	0.9676	0.9668	0.9347	0.9337
	Std Dev = 0.70	0.9289	0.9339	0.9309	0.9289	0.8648	0.8582
FGSM adversarial attack	Epsilon = 0.02	0.9621	0.9632	0.9614	0.9620	0.9247	0.9240
	Epsilon = 0.03	0.9479	0.9485	0.9473	0.9477	0.8959	0.8955

## 7 Explainable AI framework

Model decision-making processes need to be transparent and interpretable to be deployed in a clinical setting. The framework combines various complementary *post-hoc* diagnostic explanation methods. Gradient-weighted class activation mapping is used to visualize the important spatial areas of predictions. It computes weighted means of convolutional final feature maps with accuracy. This method is useful in bringing to the fore various cases of ulcers in images. Layer-specific class activation mapping is localized based on previous layers of a network. It produces more detailed heatmaps based on pixel-wise gradient data. The technique keeps the fine spatial limits of selected pathological tissues. Local interpretable model-agnostic explanations build local surrogate linear models with accuracy. It distorts input images by covering adjacent superpixel blocks one at a time. The resulting prediction alters the training of a simple interpretable linear model. In this model, superpixels that have the greatest impact on the final classification are identified. SHapley Additive Explanations give game-theoretic attributions of feature importance values. The implementation incorporates gradients of a reference distribution at baseline in a proper manner. This framework is able to balance high accuracy and clinical interpretability requirements.

### 7.1 Grad-CAM++

Grad-CAM++ creates predictions of critical image regions (23). It is better than standard gradient-weighted class activation mapping. This method emphasizes the areas that have the strongest impact on a particular class. It calculates weighted gradients on the end convolutional feature maps. These gradients are a measure of the significance of every location of spatial activation. The technique is especially successful in the localization of several different object instances. This is necessary in order to identify multiple tiny ulcer areas. The algorithm will first calculate the gradients of a target class score. These gradients back-propagate through the network to the final convolutional layer. The positive gradients show the characteristics that contribute to the high score of a class. The heatmap is a weighted average of these positive gradients. The weight, $w_{k}^{c}$, of a particular feature map *k* and a particular class *c* is defined by Equation (16), as follows:

$$w_{k}^{c}=\underset{i,j}{\sum }\alpha_{ijk}^{c}\cdot \mathrm{ReLU}!\left(\frac{\partial Y^{c}}{\partial A_{ijk}}\right)$$ (16)

Here, $\alpha_{ijk}^{c}\;$ denotes a weighting coefficient. This coefficient emphasizes higher-order spatial gradient contributions. The ReLU function retains only features with a positive influence. The final heatmap is a linear combination of forward activations. This combination uses the computed $w_{k}^{c}$.

**Figure 5:**
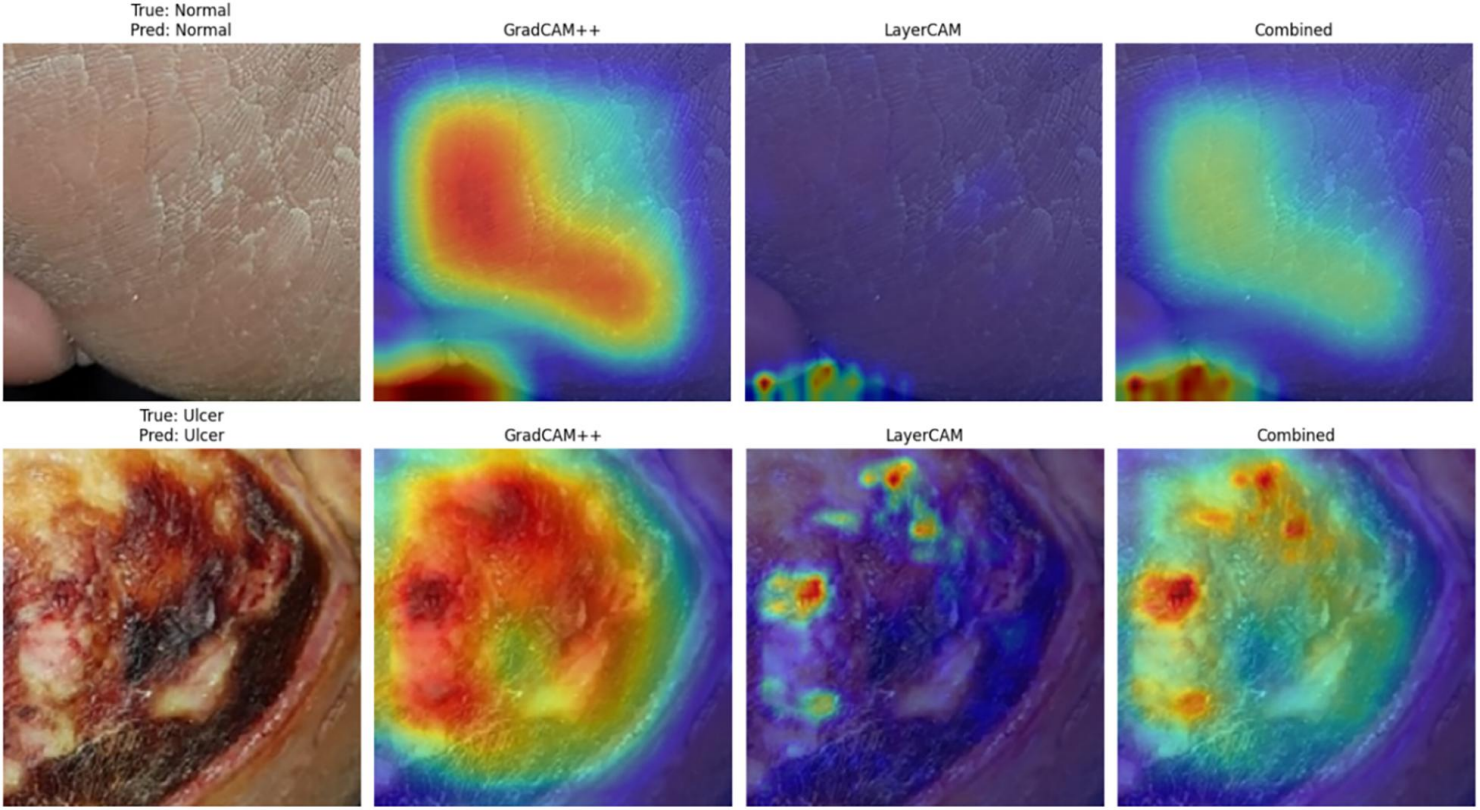
Gradient-weighted class activation mapping visualizations for ulcer localization.

The model attention mechanisms are visualized in Figure 5 with the help of Grad-CAM++. The figure contrasts the raw clinical images with the generated heatmap, where warmer colors, such as red and yellow, denote the areas of high significance. The fact that these overlays are highly concentrated on the ulcer bed and lesion boundaries instead of the irrelevant background noise makes this clear. The comparison to LayerCam also shows the localization of specific pathological features by the model. This is critical visual data for affirming clinical validation, since it demonstrates that the algorithm recognizes diabetic foot ulcers according to medically significant visual patterns, as opposed to confounding artifacts.

### 7.2 LIME

LIME constructs interpretable local surrogate models for individual predictions (24) and explains complex model decisions by perturbing the input image. The input is first segmented into meaningful superpixel regions. These superpixels represent contiguous areas with similar visual characteristics. Random perturbations are then generated by occluding different superpixel combinations. Each perturbed sample produces a corresponding model prediction probability. A simpler interpretable model is trained on these perturbed samples. This model is typically a sparse linear regression or classifier. The training data consists of perturbed binary vectors and prediction changes. Each sample is weighted by its proximity to the original input. Proximity is measured using a kernel function on the feature space. The interpretable model approximates the complex model's local behavior.

The objective function for learning the explanation is defined in Equation (17) as follows :

$$\xi (x)=\arg\underset{g\in G}{\min}L(\;f,g,\pi_{x})+\Omega (g)$$ (17)

Here, *x* represents the original image requiring an explanation. The function *f* is the original complex prediction model. The surrogate model *g* belongs to a class of interpretable models *G*. The loss *L* measures how well *g* approximates *f* locally. The weighting kernel *π_x_* assigns higher importance to nearby perturbations. The regularization term Ω(*g*) encourages explanation sparsity and simplicity. The resulting linear model coefficients indicate superpixel importance. Positive coefficients signify regions that support the predicted class. Any negative value in the coefficients shows the areas where the decision of the model is not followed. This gives a human comprehensible explanation of each particular prediction. LIME, in this way, increases trust and transparency to clinical end users.

**Figure 6:**
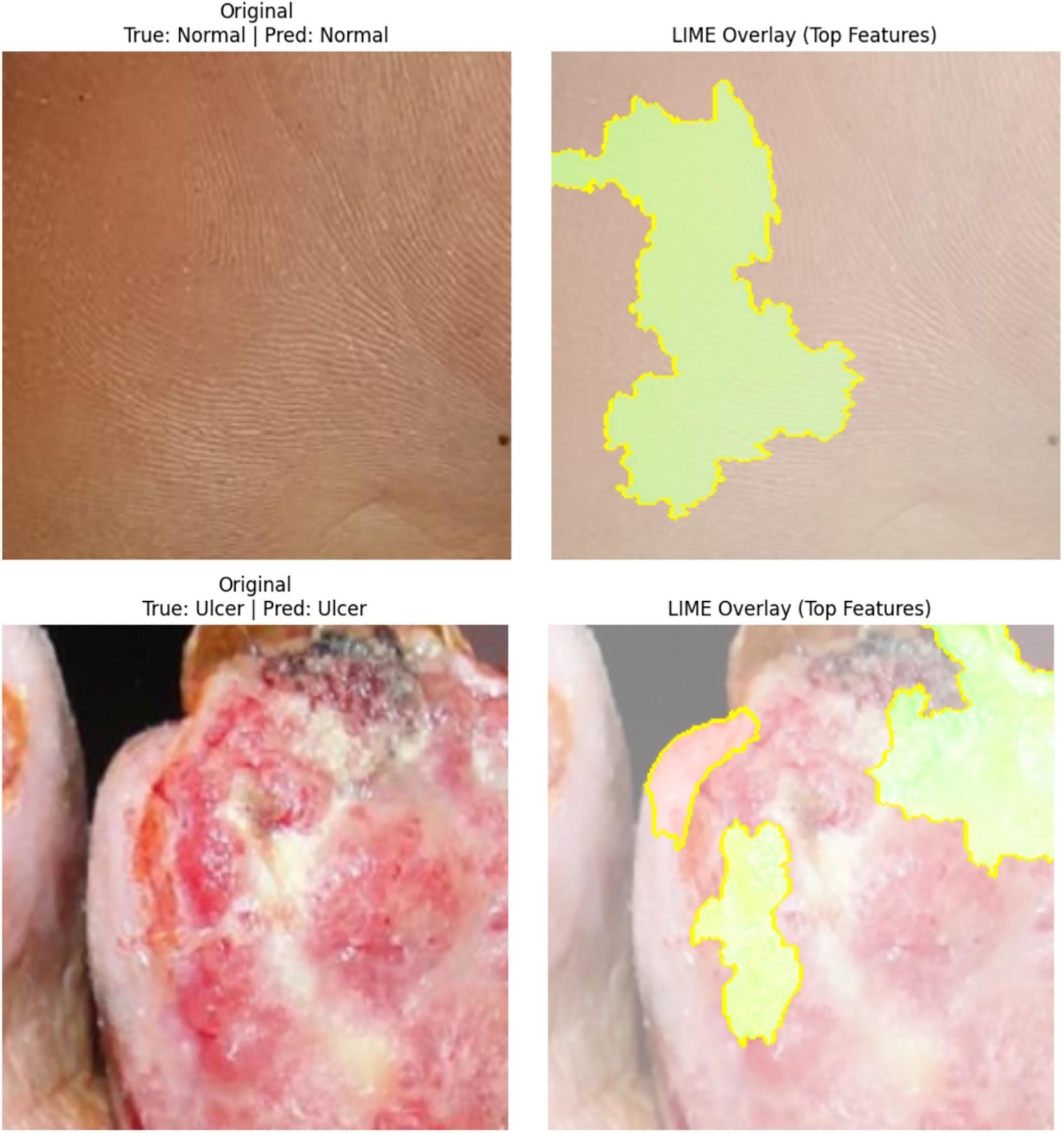
Local interpretable model-agnostic explanations demonstrating superpixel feature importance.

Local interpretable model-agnostic explanations are used to illustrate the interpretability of the model in Figure 6. The figure shows clinical images divided into adjacent superpixels according to visual similarity. Yellow markings indicate the individual superpixels that have a positive impact on the prediction of an ulcer by the model. Tracing the morphological features causing the diagnosis, the figure was obtained by disrupting these areas and examining the resulting changes in the prediction. The granular insight offered by this local explanation method enables clinicians to ensure that the model is properly identifying the location of the wound and the surrounding tissue discoloration as the main point on which the classification should be made.

### 7.3 SHAP

SHAP gives a game-theoretic framework of model interpretability (25) and assigns the output prediction to every single input feature using the cooperative game theory Shapley values approach. Each feature is regarded as a participant in a joint game. The prediction is the total payout to be distributed among players. The Shapley value calculates a feature's average marginal contribution. This contribution is averaged over all possible feature coalitions.

For a given model *f* and instance *x*, the SHAP explanation is given by Equation (18) as follows:

$$\varphi_{i}(\;f,x)=\underset{S\subseteq N\setminus \{i\}}{\sum }\frac{|S|!\;(|N|-|S|-1)!}{|N|!}[\;f(S\cup \{i\})-f(S)]$$ (18)

Here, *N* is the set of all input features and *i* is a specific feature. The term *S* represents a subset of features excluding *i*. The model *f*(*S*) denotes the prediction using only the feature subset *S*. The difference f(S∪{i})−f(S) is the marginal contribution. The weighting term accounts for all possible ordering permutations.

The total of all feature attributions is equal to the model’s deviation. This is off-target from the desired model performance. The property guarantees local accuracy and consistency in explanations. Deep learning models can be estimated in terms of SHAP with a high degree of efficiency. The GradientExplainer technique combines gradients in a reference path baseline. This baseline tends to utilize a reference of normal healthy skin samples. The last explanation gives the pixels that back up the prediction or refute it. It offers global and local interpretability in order to validate clinical performance. SHAP therefore provides mathematically rigorous and consistent feature attribution.

**Figure 7:**
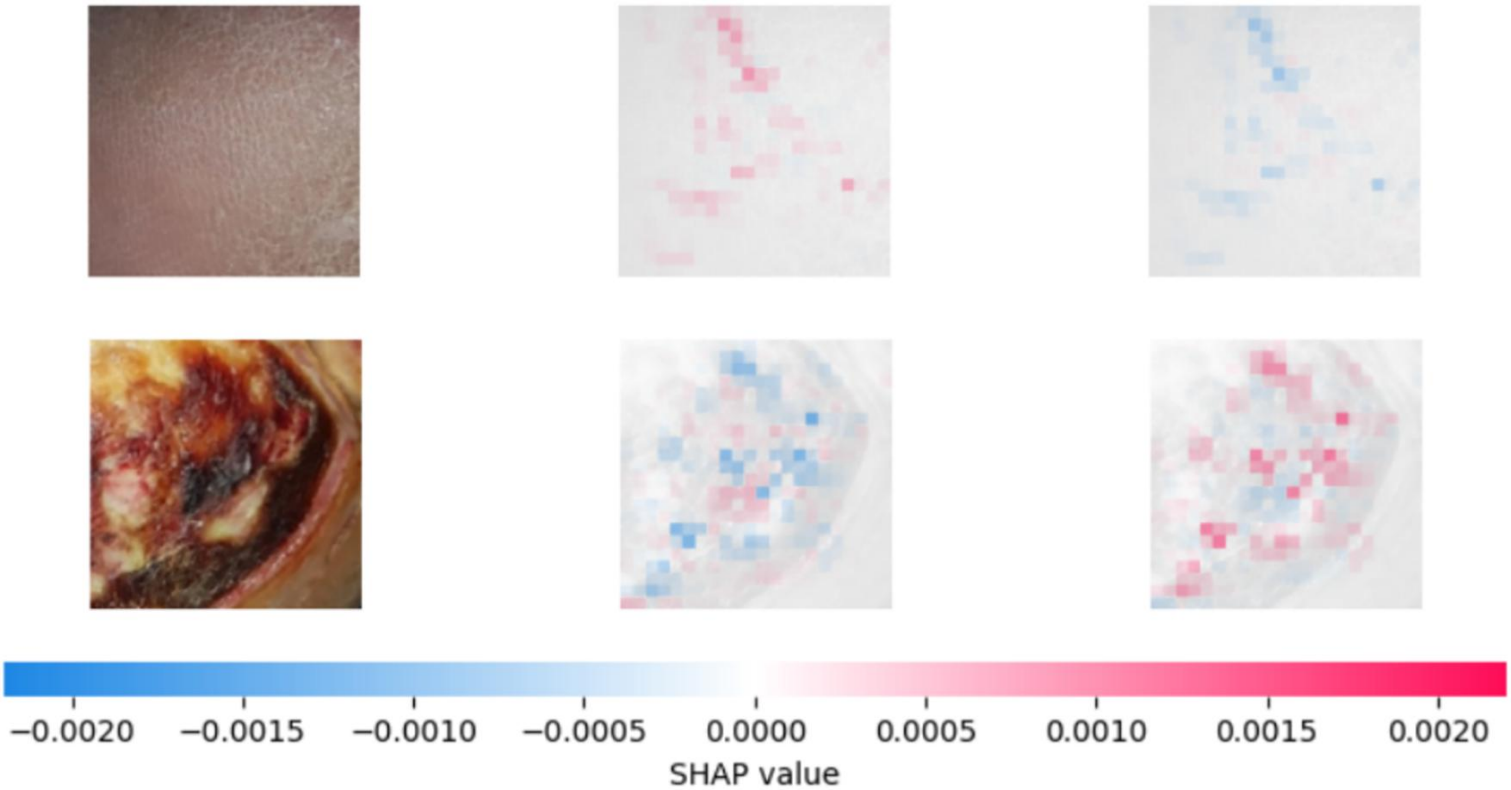
SHAP summary plots highlighting pixel-level contributions to predictions.

In Figure 7, SHapley Additive exPlanations are used to assign prediction contributions on a pixel-level. The plot shows input samples and maps of the SHAP values, which were obtained with the help of the GradientExplainer method. The red pixels represent features that are likely to give an ulcer diagnosis, whereas blue pixels indicate healthy skin. This game-based model guarantees the mathematical consistency of the importance of the feature distribution. The ensuing visualizations affirm that the model identifies fine-grained textural abnormalities and color differences related to pathology. This process subjects the model to a strict test of logic, which provides transparency and confidence among medical practitioners.

## 8 Limitations and future work

While the proposed DFU-GCNet demonstrates state-of-the-art predictive capability, this study is constrained by certain limitations that warrant discussion. Foremost is the restricted volume of the dataset (1,055 instances). In the medical domain, acquiring massive, expertly annotated cohorts is frequently prohibited by strict privacy regulations and prohibitive annotation costs. While we have mathematically justified the model's resilience against overfitting through rigorous five-fold cross-validation and comprehensive regularization, the relatively small sample size fundamentally limits the ultimate breadth of pathological variance the network can assimilate.

Furthermore, the dataset exhibits inherent geographic and demographic bias, as all clinical images were sourced from a single institution. Consequently, the model's feature exposure is restricted to a specific demographic profile, which inherently constrains its immediate zero-shot generalization capabilities to phenotypically diverse global populations. Additionally, the reliance on specific consumer-grade hardware introduces a subtle hardware-induced domain bias.

Beyond data-centric constraints, translating this framework into a functional clinical tool entails substantial real-world deployment challenges. Operational clinical integration necessitates seamless interoperability with existing electronic health record systems and strict adherence to medical regulatory frameworks. Moreover, in unconstrained clinical settings, diagnostic models must reliably process out-of-distribution visual artifacts, such as diverse ambient illumination, the presence of surgical instruments, or occluding bandages, without suffering catastrophic drops in predictive confidence. Additionally, deploying such high-parameter architectures at the clinical edge will require aggressive model quantization and computational optimization.

To rigorously establish cross-domain generalization and address these limitations, our immediate future work will involve testing and cross-validating the DFU-GCNet architecture on independent, external benchmark datasets. Subsequent research will also prioritize multi-center federated learning to aggregate geographically diverse data without compromising patient privacy. Future iterations of the framework will expand beyond binary detection to incorporate multi-class grading of tissue necrosis, integrate depth-sensing for volumetric wound assessment, and leverage multimodal clinical histories, thereby evolving the current architecture into a highly robust, clinically deployable diagnostic pipeline.

## 9 Conclusion

This study presents DFU-GCNet for automated diabetic foot ulcer detection. The model combines multi-scale inception modules with global context attention. This architecture successfully captures highly complex ulcer morphologies. It extracts local textures and models global dependencies effectively. Empirical tests on the Kaggle dataset confirm its robustness. Our optimized framework achieved an outstanding test accuracy of 98.12% and a Matthews correlation coefficient of 0.9631. The *F*1-score reached 0.9801 with a Kappa score of 0.9622. These metrics conclusively outperformed established baselines such as VGG16 and ResNet18. Our model handles medical class imbalance with exceptional proficiency. Furthermore, we integrated a comprehensive explainable AI suite. Grad-CAM++, LIME, and SHAP were used to validate the model. Visualizations confirmed the network focuses strictly on lesion boundaries. It consistently ignores irrelevant background artifacts during classification. This transparency is crucial for building vital clinical trust. Despite these successes, the current scope remains binary classification. Future research will explore multi-class grading of infection severity. We also aim to deploy this in mobile healthcare settings.

## Data Availability

The original contributions presented in the study are included in the article/Supplementary Material, further inquiries can be directed to the corresponding author.
